# Supplementary material for: Self-implantable double-layered micro-drug-reservoirs for efficient and controlled ocular drug delivery
Source: Nat Commun. 2018 Nov 6;9:4433. doi: 10.1038/s41467-018-06981-w (PMC6219513; doi:10.1038/s41467-018-06981-w)
Supplement: Supplementary file 1 — Supplementary Information [file 41467_2018_6981_MOESM1_ESM.pdf]

Self-implantable double-layered micro-drug-reservoirs for efficient and controlled ocular drug delivery

A. Than *et al*.

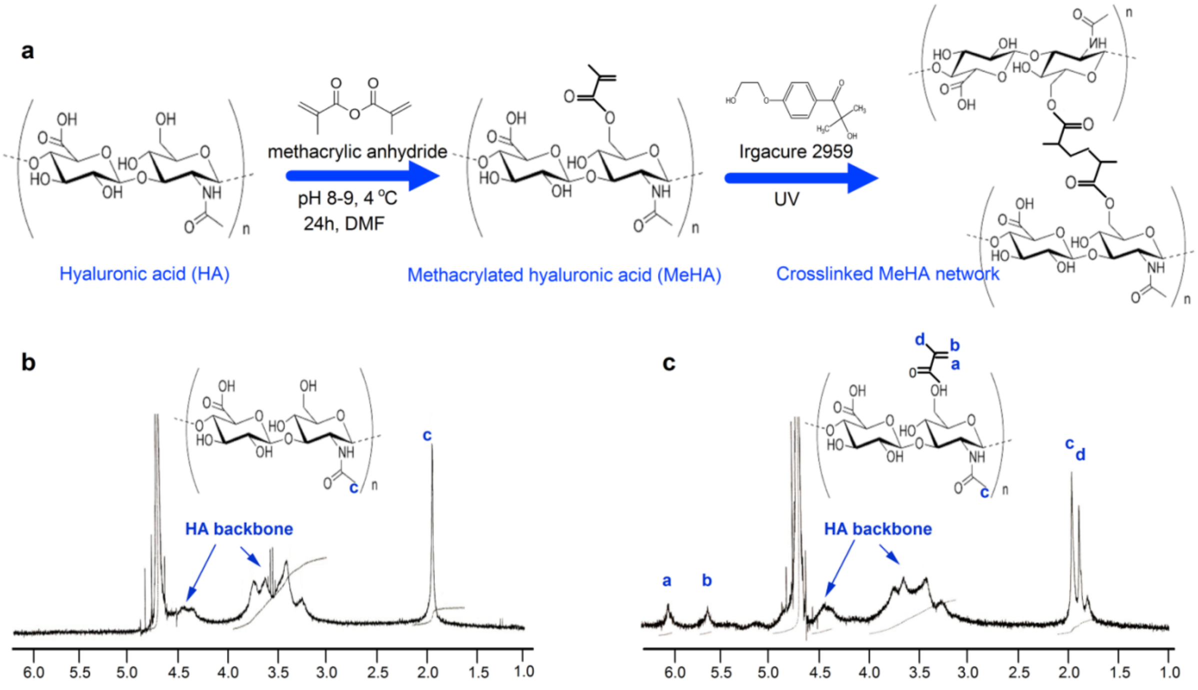

Supplementary Figure 1: Synthesis and characterization of methacrylated hyaluronic acid (MeHA). (a) Schematic of the synthesis of MeHA and the crosslinking through photo-activation. Briefly, N,N-dimethyl-formamide (133.3 ml) and methacrylic anhydride (4.7 ml) were added dropwise to HA solution (4.0 g of ~300 kDa HA in 200 ml DI water), and adjusted to pH 8 - 9 with sodium hydroxide (NaOH). After continuous stirring for 1 day (4 °C), the reaction solution was supplemented with sodium chloride (NaCl, 9.88 g) to precipitate MeHA in ethanol. MeHA precipitates were washed again with ethanol for 3 times, dissolved in DI water and dialyzed for 7 days. After lyophilisation, the purified MeHA was characterized by ^{1}H NMR spectroscopy using a Bruker Avance II NMR (300MHz, D2O, room temperature). The degree of modification was determined by digital integration of the anomeric protons signals or methyl protons signals of HA and of the methacrylate proton signals at ~6.1, ~5.7, and ~1.9 ppm. The degree of methacrylation was ~70% according to the ^{1}H NMR map. **(b and c)** show representative ^{1}H NMR spectra of HA and MeHA.

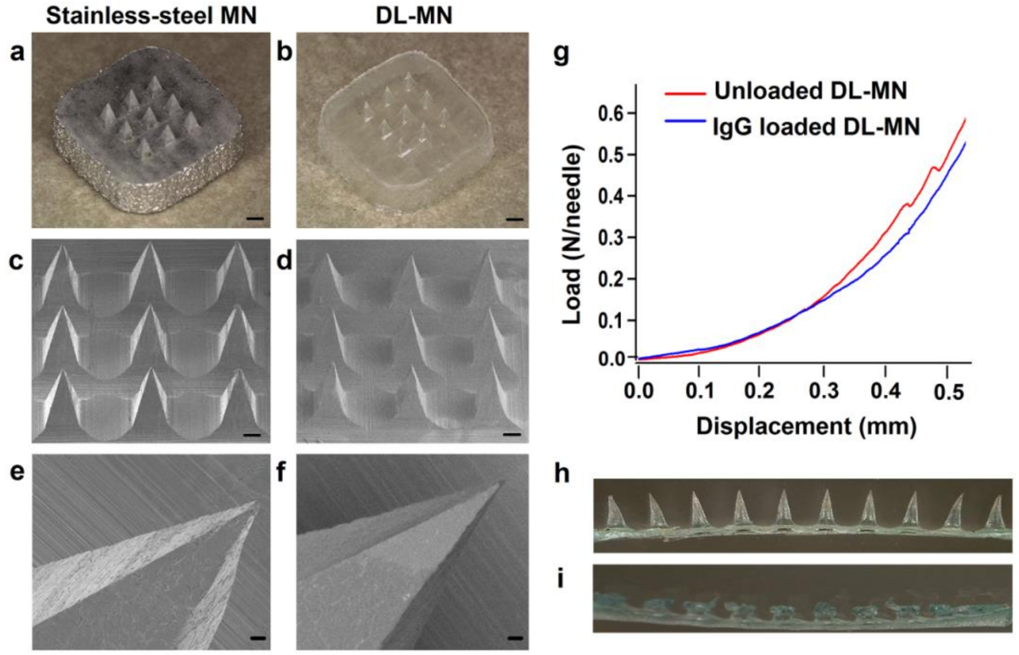

Supplementary Figure 2: Characterization of double-layered microneedles. (a - b) Bright-field and **(c - f)** scanning-electron microscopy images of stainless-steel MN master-mold and corresponding DL-MN patch; Scale bars = 400 µm (a and b), 100 µm (c and d) or 10 µm (e and f). **(g)** Mechanical compression test (average from 4 measurements) of un-loaded (red line) and IgG(680)-loaded DL-MN (blue line) (2 µg in 9 MNs). **(h and i)** Bright-field images of DL-MNs, (h) before and (i) after compression test.

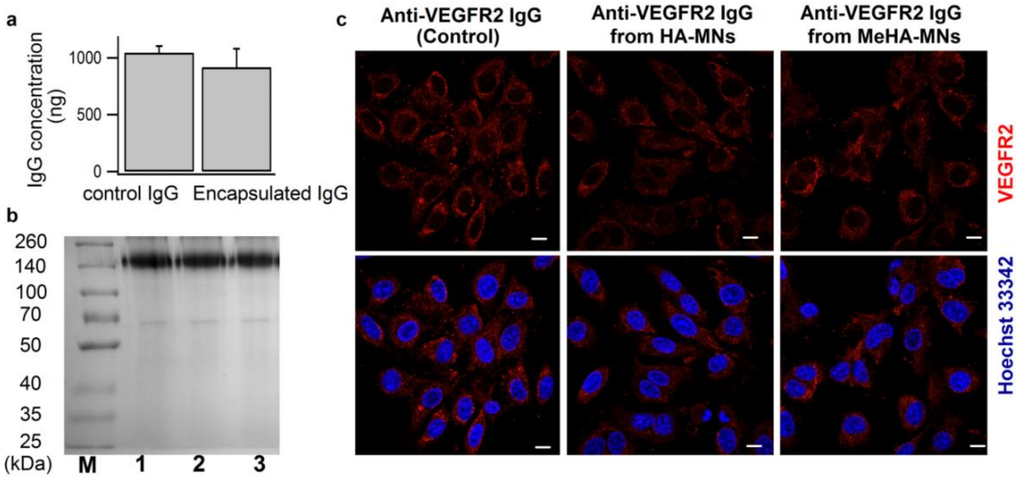

Supplementary Figure 3: In vitro studies of double-layered microneedles loaded with anti-VEGFR2 IgGs. (a) Quantification of anti-VEGFR2 IgGs loaded in 3 x 3 arrays of DL-MNs using an Easy-Titer IgG assay kit (ThermoFisher Scientific). 1 µg of IgG equally divided into the inner core and outer shell of MN. Data represent mean ± SEM (3 samples for each group). **(b)** Protein staining on 12% polyacrylamide gel loaded with anti-VEGFR2 IgGs. Lane 1: freshly-prepared IgG; lane 2: IgG collected from HA-MN; lane 3: IgG collected from

MeHA-MN; lane M: molecular weight markers. Protein bands were stained with InstantBlue solution (Expedeon) and detected in a G:BOX Chemi XT4 imaging system (Syngene). **(c)** The representative confocal images of immunostained VEGFR2 in primary human endothelial cells (HUVECs), using the freshly-prepared anti-VEGFR2 IgG or anti-VEGFR2 IgG released from HA-MN / MeHA-MNs (being stored for 5 days) over 24 hr duration. Hoechst 33342 (NucBlue Live ReadyProbes Reagent, Life Technologies) was used to stain the nuclei. The red fluorescence indicates the staining of VEGFR2 (Alexa Fluor 680). Scale bars = 10 μm.

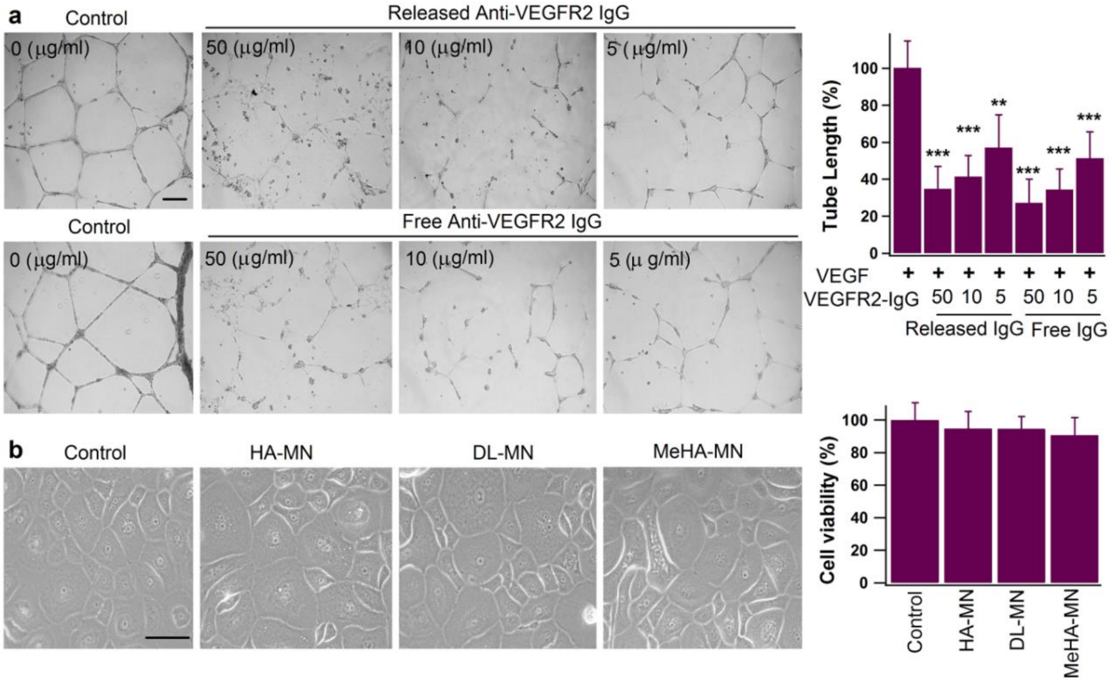

Supplementary Figure 4: In vitro studies of double-layered microneedles. (a - d) In vitro anti-angiogenic activity of anti-VEGFR2 IgG in primary human endothelial cells (HUEVCs). **(a)** Anti-VEGFR2 IgG released from DL-MNs (being stored for 5 days) over 6 hr (5 μg/ml), 24 hr (10 μg/ml) or 120 hr (50 μg/ml) duration was used to treat the cells for ~18 hr (with 10 ng VEGF). Freshly prepared IgG at different concentrations was also tested for comparison. Representative bright-field images (left) of tube formation in Matrigel and the statistics (right) of tube length (%control) are shown (n = 4). Scale bar = 100 μm. **(b)** In vitro biocompatibility of MNs in primary human corneal epithelial cells. Cells (Merck Millipore, SCCE016) were exposed to different types of MNs for 2 days. Representative bright-field images and the statistics of cell viability (%control) (using the AlamarBlue assay) are shown (n = 4). Scale bar = 50 μm. The data represents mean ± SEM. n represents the number of samples for each group. Statistical comparison between groups was performed using Student's *t* test. $**p < 0.01$ and $***p < 0.005$.

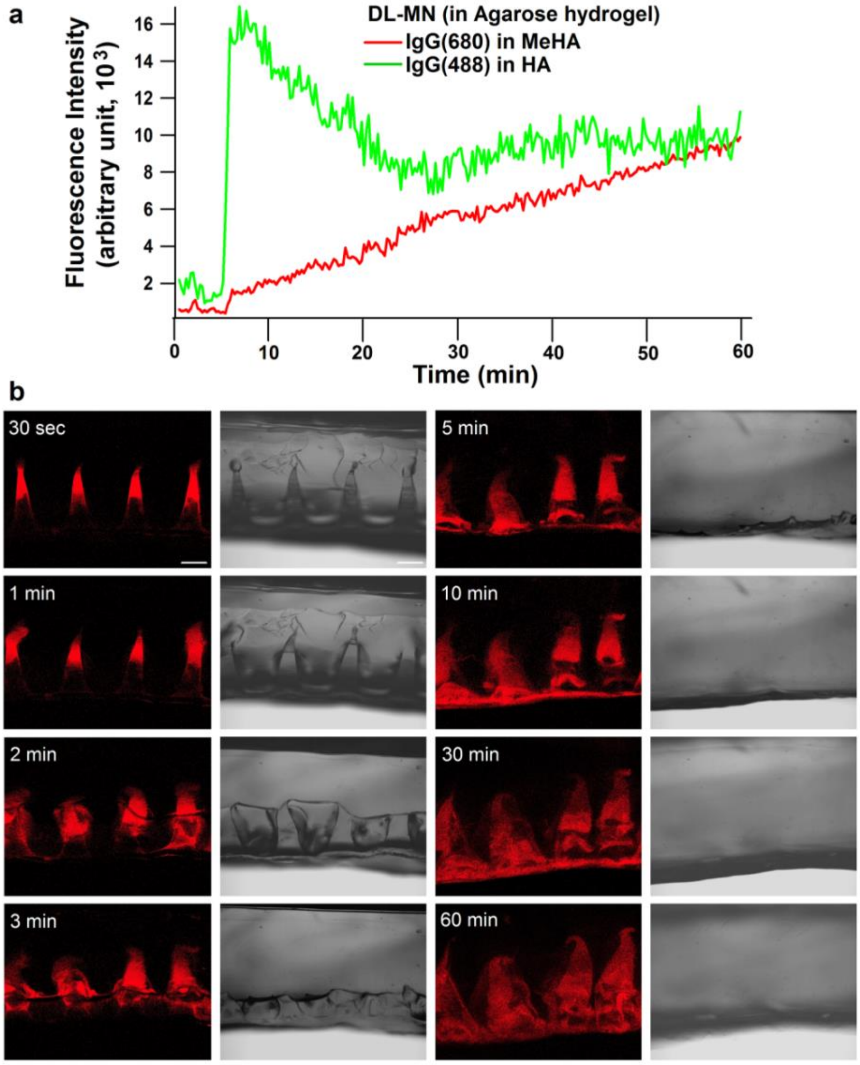

Supplementary Figure 5: Biphasic release profile of double-layered microneedles. (a) The average changes of fluorescence intensity (average from 4 measurements) in the region adjacent to DL-MNs due to released IgG(680) from the MeHA outer layer (red line) and IgG(488) from the HA inner core (green line). **(b)** Representative time-lapse confocal images and corresponding bright-field images of DL-MN in agarose hydrogel, showing the slow-release of IgG(680) from the outer layer of DL-MN. Note that DL-MN become clear and transparent after 2 min. Scale bar = 200 μm.

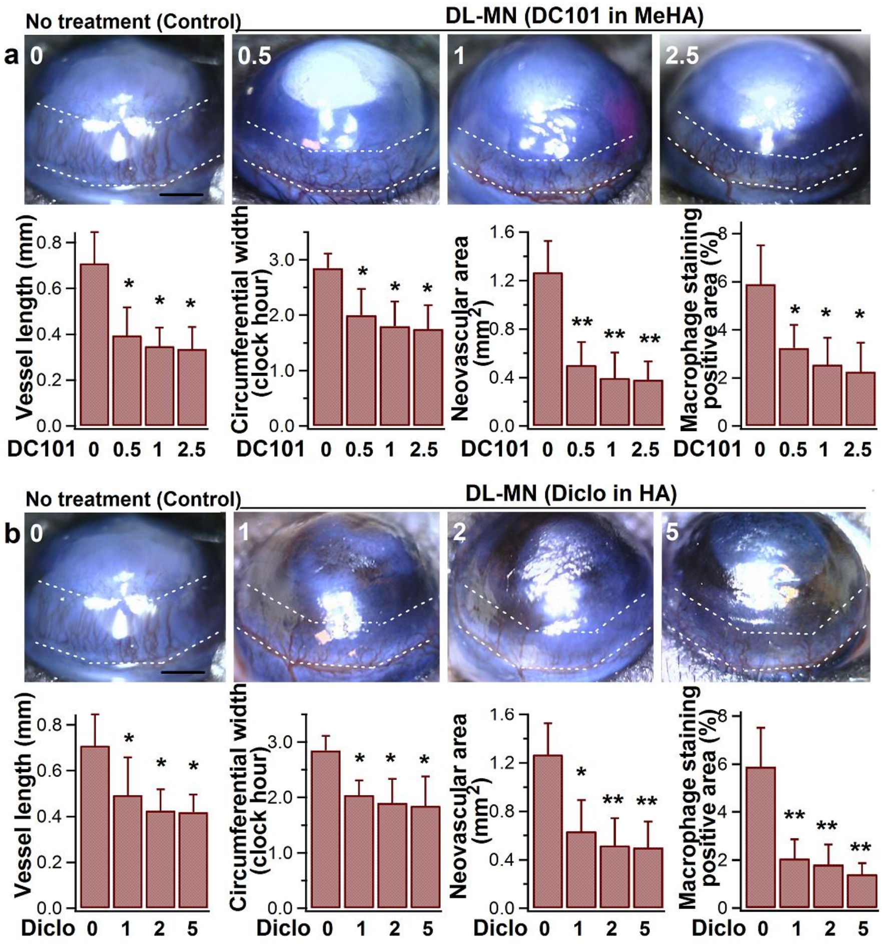

Supplementary Figure 6: Double-layered microneedle patch for ocular drug delivery. (a) DC101 (0, 0.5, 1 or 2.5 µg in outer layer of MeHA) or **(b)** Diclofenac (Diclo) (0, 1, 2 or 5 µg in inner core of HA) were loaded in DL-MN. Mouse eyes were then treated differently 2 days after being inflicted with alkali-burn, and examined at day 7. Representative images of differently treated eyes and quantifications of corneal neovascularization (mean ± SEM; 4 samples for each group) are shown. The white dotted lines indicate the extent of neovascular outgrowth from the limbus. Quantifications of macrophage accumulation (% of positive staining of a specific macrophage marker - F4/80) in differently treated eyes (mean ± SEM; 4 samples for each group) are also shown. Scale bars = 500 µm. Statistical comparison between groups was performed using one way ANOVA. *$p < 0.05$, **$p < 0.01$ vs. control.

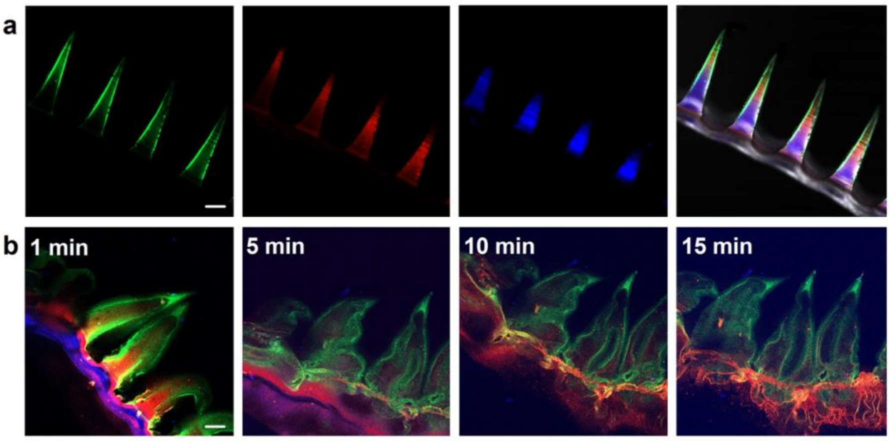

Supplementary Figure 7: Tri-layered microneedles for multi-drug delivery. (a) The representative confocal image of triple-layered MN, with the outer layer made of crosslinked MeHA containing IgG(488) (green colour), middle layer made of HA (~50 kDa) containing IgG(680) (red colour), and inner core made of HA (<10 kDa) containing IgG(405) (blue colour). **(b)** Time-lapse confocal images of real-time release from triple-layered MN in agarose hydrogel, showing rapid release of IgG(405), followed by slower release of IgG(680), and slowest release of IgG(488). Scale bars = 200 µm.

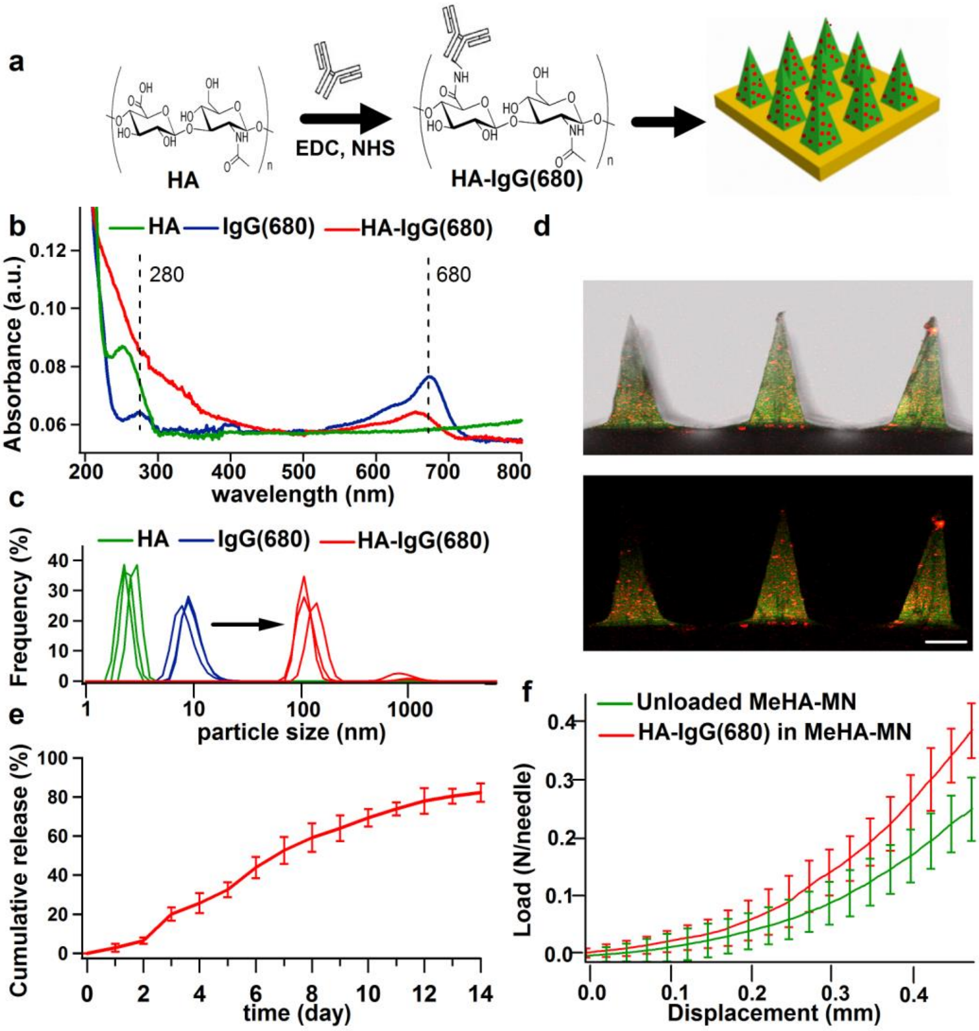

Supplementary Figure 8: Prolonged release of HA-IgG conjugates loaded in microneedles. (a) Schematic of HA and IgG conjugation, and loading into MeHA-MN. **(b)** UV-Vis spectra of HA (green line), IgG(680) (blue line) and HA-IgG(680) conjugate (red line) confirm the success of conjugation. **(c)** Size distribution of HA (green line), IgG(680) (blue line) and HA-IgG(680) (red line) determined by dynamic light scattering (DLS) analyses indicate that HA-IgG(680) forms larger nanoparticles. **(d)** Representative confocal image of MeHA-MN loaded with HA-IgG(680) conjugates. Scale bar = 200 µm. **(e)** In vitro release profiles of HA-IgG(680) from MeHA-MN in PBS ($t_{1/2}$ of ~1 week) (n = 3). **(f)** Mechanical compression test of unloaded (green line) or HA-IgG(680) (red line) loaded MeHA-MN (n = 4). Data represents the mean ± SEM. n represents the number of samples for each group.
